# An Open-Source, Automated Home-Cage Sipper Device for Monitoring Liquid Ingestive Behavior in Rodents

**DOI:** 10.1523/ENEURO.0292-19.2019

**Published:** 2019-10-08

**Authors:** Elizabeth Godynyuk, Maya N. Bluitt, Jessica R. Tooley, Alexxai V. Kravitz, Meaghan C. Creed

**Affiliations:** 1 Department of Anesthesiology, Washington University in St. Louis, St. Louis, Missouri 63108; 2 Department of Psychiatry, Washington University in St. Louis, St. Louis, Missouri 63108; 3 Departments of Neuroscience and Biomedical Engineering, Washington University in St. Louis, St. Louis, Missouri 63108

**Keywords:** Arduino, open source hardware, two-bottle choice

## Abstract

Measuring ingestive behavior of liquids in rodents is commonly used in studies of reward, metabolism, and circadian biology. Common approaches for measuring liquid intake in real time include computer-tethered lickometers or video-based systems. Additionally, liquids can be measured or weighed to determine the amount consumed without real-time sensing. Here, we built a photobeam-based sipper device that has the following advantages over traditional methods: (1) it is battery powered and fits in vivarium caging to allow home-cage measurements; (2) it quantifies the intake of two different liquids simultaneously for preference studies; (3) it is low cost and easily constructed, enabling high-throughput experiments; and (4) it is open source so that others can modify it to fit their experimental needs. We validated the performance of this device in three experiments. First, we calibrated our device using time-lapse video-based measurements of liquid intake and correlated sipper interactions with liquid intake. Second, we used the sipper device to measure preference for water versus chocolate milk, demonstrating its utility for two-bottle choice tasks. Third, we integrated the device with fiber photometry, establishing its utility for measuring neural activity in studies of ingestive behavior. This device requires no special equipment or caging, and is small, battery powered, and wireless, allowing it to be placed directly in rodent home cages. The total cost of fabrication is less than $100, and all design files and code are open source. Together, these factors greatly increase scalability and utility for a variety of behavioral neuroscience applications.

## Visual Abstract

**Figure:**
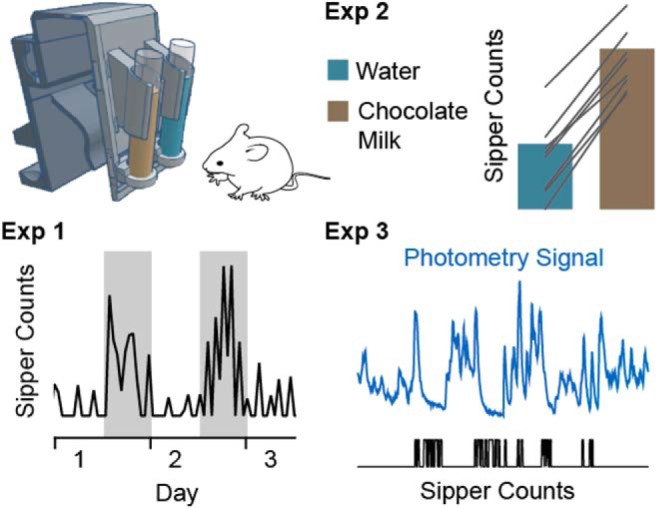

## Significance Statement

Current methods for measuring liquid consumption in rodents typically require specialized equipment that is tethered to an in-room computer. This makes portability and scalability challenging. We present a device that is small, battery powered, and wireless, allowing it to be placed directly in rodent home cages. Moreover, the total cost of fabrication is less than $100 and the design is open source. Together, these factors greatly increase scalability, as the devices do not require dedicated experimental space or caging. The battery lasts ∼2 weeks, enabling studies of long-term intake or circadian biology.

## Introduction

Studies of liquid ingestive behavior are widely used in rodent studies of reward-related behavior, metabolism, and circadian biology. Most directly, measurements of liquid intake can be used to understand thirst, a powerful motivational state that is critical for survival and is regulated by metabolic factors (Ho and Chin, 1988; Greenleaf, 1994; Camandola and Mattson, 2017). Tracking liquid intake is a passive way of determining circadian cycle (Schwartz and Zimmerman, 1990) and of monitoring the effect of genetic or pharmacological manipulations on circadian biology (Ho and Chin, 1988; Mistlberger et al., 2001; Bainier et al., 2017). Moreover, drinking water is a common route of drug administration in behavioral pharmacology studies (Hsiao and Spencer, 1983; Genn et al., 2003), requiring detailed measurements and a temporal profile of drinking water consumed to determine drug dosage. Finally, by examining the relative consumption of two fluids, the “preference” can be determined, which relates to a specific motivation for one fluid over another. The strength of a preference for sucrose has been used as a measure of anhedonia (the inability to feel pleasure) in animal models of depression and drug withdrawal (Eagle et al., 2016; Alkhlaif et al., 2017; Liu et al., 2018), while alcohol or drug preference assays (Shaham et al., 1992; Ackroff et al., 1993; Adriani et al., 2002; Hwa et al., 2011; Ackroff and Sclafani, 2013) or liquid self-administration are widely used in addiction neuroscience (Peachey et al., 1976; Shaham et al., 1992; Isiegas et al., 2009).

Existing methods of measuring liquid intake fall into two broad categories. The first is the “low-tech” method of placing a graduated tube in the cage and visually inspecting it to determine how much liquid was consumed (Tordoff, 2007; Tordoff et al., 2007). This method is widely used as it is inexpensive, robust, and does not require specialized equipment or caging. However, this method does not reveal information on the microstructure of licking or changes in the temporal patterns of ingestive behavior. The second is a more “high-tech” method, which involves placing animals in specialized caging that automatically monitors liquid intake over time, for example by continuously sampling the weight of a water bottle or by registering optical or electrical contacts with a lickometer (for review, see Weijnen, 1998). This approach is automatic and provides temporal resolution, but requires wiring (Hayar et al., 2006) or expensive, specialized equipment and caging, which limit its accessibility and applicability for high-throughput or long-term studies. Moreover, electrical lickometers that rely on capacitative sensing can produce electrical artifacts that limit their use with some *in vivo* measures of neuronal activity (Schoenbaum et al., 2001; Hayar et al., 2006).

Here, we provide detailed instructions for the construction of a novel, open-source, home cage-based sipper device, which has all the functionality of the previously described low-tech methods, and has several advantages over existing high-tech methods. Our device was developed with scalability in mind and, as such, was designed to be low cost (under $100), wireless, and easy to build by users with no previous electronics experience. The device fits in traditional vivarium home cages, facilitating the study of large populations without requiring additional experimental space or equipment beyond the vivarium racks. The device is also battery powered, enabling continuous data collection over 2 weeks with minimal experimenter interference. Finally, since the device uses optical sensors (photo-interrupters) rather than capacitative sensing to detect interactions with each sipper, it will not introduce electrical artifacts into data acquisition, and is thus compatible with amplifiers for *in vivo* electrophysiology, fiber photometry systems, or behavioral equipment capable of receiving a transistor-transistor logic (TTL) input.

We validated the sipper device across three experiments. First, we validated the sipper devices as a proxy of ingestive behavior and observed a clear circadian rhythm of drinking water consumption. Second, we used the sipper in a two-bottle choice assay and determined that the device can reliably detect preference for a preferred liquid (chocolate milk) over water. Finally, we performed a proof-of principle demonstration that the sipper device can easily and successfully integrate with a fiber photometry system, establishing its utility in studying the neural substrate of ingestive behavior. Together, these validation experiments confirm that the sipper device can be used to measure the ingestive behavior of liquids across multiple behavioral neuroscience paradigms.

## Materials and Methods

### Animals and experimental procedures

A total of 24 adult mice (C57BL/6J background; age, 10–16 weeks) were housed in standard mouse vivarium caging and kept on a 12 h light/dark cycle (light onset, 7:00 A.M.; light offset, 7:00 P.M.) with *ad libitum* access to food and water. Both male and female mice were included in the experiments (14 male, 10 female). Mice were single housed after surgery to protect cranial implants. All studies were approved by the internal animal care and use committee at Washington University in St. Louis.

#### Experiment 1

To characterize how the counts and duration of the sipper device reflect the volume of liquid consumed, mice (7 male, 5 female) were individually housed, and sipper devices were run overnight as the sole source of drinking water in the home cage. Videos were taken overnight using a time-lapse camera (TLC200, Brinno) and exported, and the position of meniscus in the conical tube reservoir of the sipper device was registered using ImageJ to visually validate the amount of liquid consumed at 1 h intervals. As an extension, we ran this experiment overnight for 6 d to determine whether the sipper device detected circadian patterns of activity. The mice used in these validation studies had not undergone any prior surgeries nor had they been used for previous experiments.

#### Experiment 2

To confirm that the sipper device can robustly detect the ingestion of two liquids simultaneously, mice (4 male, 5 female) were individually housed, and given access to both drinking water and chocolate milk (diluted 50% with drinking water) via the sipper device for a 4 h “two-bottle choice” assay.

#### Experiment 3

To confirm that the device can be successfully integrated with *in vivo* measurements of neural activity (using population calcium activity as a proxy), we expressed the calcium indicator GCaMP6s (AAVDJ-hsyn-GCaMP6s-eYFP, University of North Carolina Vector Core) in the dorsomedial striatum (DMS; coordinates from bregma: anteroposterior, +0.5 mm; mediolateral, ±1.5 mm; dorsoventral, −2.8 mm; according to the Atlas of Franklin and Paxinos) and implanted a fiber (200 µm core, 0.39 numerical aperture; ThorLabs) in the same region in wild-type mice (*n* = 3 males).

Mice were allowed to consume chocolate milk from the sipper device while GCaMP fluorescence was detected through the optic fiber using a fiber photometry system (Neurophotometrics), with all data recorded in the open-source visual programming language Bonsai (Lopes et al., 2015). We aligned the photobeam breaks detected with the sipper device to the fiber photometry recording in Bonsai using an Arduino Uno as a digital acquisition device. Support for using the Arduino Uno in this manner is a native feature of Bonsai and required only a minimal modification to the sipper to solder a Bayonet Neill Concelman connector to the sipper device.

### Build instructions

**Figure 1.**
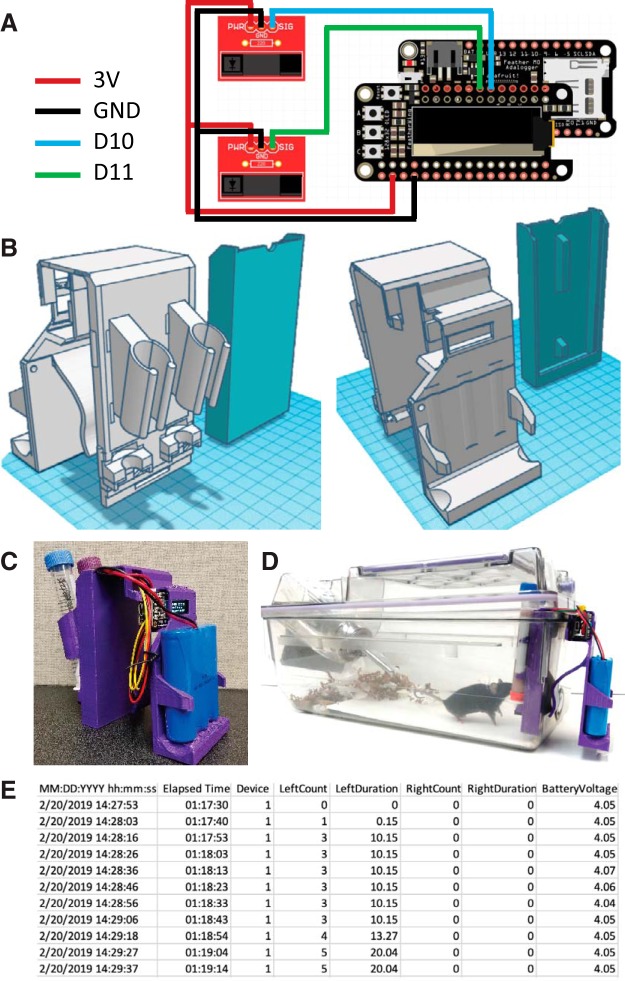
Construction and implementation of a home-cage drinking monitor. ***A***, Circuit wiring diagram of electronic components; 3 V power is supplied to the photo-interrupters, which are connected to a ground pin. Each photo-interrupter is also attached to a digital output pin (shown as D9 and D10). ***B***, 3D rendering of the 3D printed housing for the drinking monitor; views of the front tube assembly (left) and rear battery casing (right) are shown. ***C***, ***D***, Photo of the assembled device (***C***) and the assembled device operating in the home cage (***D***). ***E***, Example data as written to the SD card.

Here, we present a compact, battery-powered, wireless, home cage-compatible sipper device (Fig. 1). The device uses readily available building materials (Table 1), prioritizing “off-the-shelf” electronics such as the Adafruit Feather M0 Adalogger microcontroller (Fig. 1*A*) and 3D printed components (Fig. 1*B*). The sipper device uses infrared photo-interrupters to sense when the animal interacts with each sipper spout and displays information on a built-in screen to provide real-time data to a user, such as time spent interacting with each individual sipper as well as the number of approaches. The Feather M0 Adalogger also records this information at a user-defined frequency to an on-board microSD card (the default code is set to 6 samples/min). The device is small, battery powered, and wireless, which makes it compatible with standard rodent vivarium caging (Fig. 1*C*,*D*) and has a battery life of >2 weeks, allowing for long-term monitoring with minimal experimenter interference.

**Table 1.** Bill of materials

Component	Number	Cost / unit	Total cost	Source of materials
Adafruit Feather M0 Adalogger	1	$19.95	$19.95	https://www.adafruit.com/product/2796
Lithium Ion Battery Pack - 3.7V 6600mAh	1	$29.50	$29.50	https://www.adafruit.com/product/353
Adafruit FeatherWing OLED – 128x32 OLED	1	$14.95	14.95	https://www.adafruit.com/product/2900
Short Headers Kit for Feather -12-pin + 16-pin Female Headers	1	$1.50	$1.50	https://www.adafruit.com/product/2940
1k Resistor	2	$0.20	$0.40	https://www.sparkfun.com/products/14492
Photo Interrupter -GP1A57HRJ00F	2	$2.50	$5.00	https://www.sparkfun.com/products/9299
SparkFun Photo Interrupter Breakout Board -GP1A57HRJ00F	2	$1.50	$3.00	https://www.sparkfun.com/products/9322
MicroSD card	1	$6.00	$6.00	https://www.amazon.com/Kingston-microSDHC-Class-Memory-SDC4/dp/B00200K1TS/
JST cables	2	$1.50	$3.00	https://vetco.net/products/jst-ph-connector-male-female-pair-pre-wired-3-pin
Plastic valves	2	$0.50	$1.00	https://labproductsinc.com/product/hydropac-alternative-watering-system/
15 ml conical tubes	2			

All materials required for construction of the sipper device are itemized, sourcing and cost are provided.

All design files necessary to complete this build (including electronic layout/soldering instructions, Python code, and 3D printing design files) are located at: https://hackaday.io/project/160388-automated-mouse-homecage-two-bottle-choice-test-v2.

All Files for 3D printing, along with photographs and instructions for assembling the sipper devices can be found at: https://hackaday.io/project/160388-automated-mouse-homecage-two-bottle-choice-test-v2.

3D-Print the two housing components (Fig. 1). Assemble the sipper tubes. Using a razor or scalpel blade, cut ∼1cm of the cylindrical end from each 15 ml conical tube. Using hot glue, secure a Hydropac valve into the cut end and allow it to cool and dry. Check for leaks.Assemble photo-interrupters: solder the SparkFun photo-interrupter and 1K resistor into the SparkFun breakout board. Finally, solder the three-pin right-angle JST connecter with jumper wires into the photodetector.Solder the short female header pins to the Adalogger M0 board, and short male header pins to the Adafruit OLED wing. Solder the photo-interrupter wires to the OLED shield, with the black wires attached to the ground (GND), and red wires to power (3 V), and the signal wires to pins 10 and 11 (Fig. 1*A*).Assemble the device by pushing the photodetector units into the slots in the 3d housing and the Feather device into the holder (Fig. 1*C*). Slide the protective cover over the photodetectors, ensuring that the wires protrude on the top of the setup.Snap conical tubes into the housing.Insert the microSD card into the Feather devices and attach the battery pack.Set the on-board real-time clock (RTC). Flash the Adalogger with the code “SipCounter-SetClock.ino” to set the RTC. The RTC will retain the date and time as long as the device does not lose power. Alternatively, the date and time can be set on the device itself (details below).Flash the Adalogger with the “SipCounter-081118.ino” code. This is the code that controls the operation and function of the device.

### Operation instructions

Videos showing the setup and operation of the device can be at: https://hackaday.io/project/160388-automated-mouse-homecage-two-bottle-choice-test-v2.

When the device is powered up, it will start on a welcome screen.Holding down the “B” button on this screen allows for editing the date, time, and device number. These variables will be recorded with the data on the microSD card. Pressing “A” on this screen starts data recording. The number of interactions and the duration of interaction will be displayed on the screen. All data obtained will also be saved in 10 s increments (this frequency can be edited in the code; Fig. 1*E*).

### Statistical analysis

Analysis of correlation measurements and photometry experiments were performed in Python (3.7), and paired *t* tests and Wilcoxon signed rank tests were performed in Prism (version 8). A *p* value >0.05 was considered statistically significant. Nonparametric statistics were used in cases where the dataset did not meet assumptions of normality.

## Results

We performed three experiments to validate the use of the device in multiple paradigms relevant for behavioral neuroscience. We first validated that the device accurately tracked the volume of liquid ingested from the sipper device. We next demonstrated that the sipper device can be used to detect preference in a two-bottle choice assay. Finally, we establish that the sipper device can be simply integrated with *in vivo* measurements of neuronal activity, in this case, fiber photometry.

### Experiment 1

**Figure 2.**
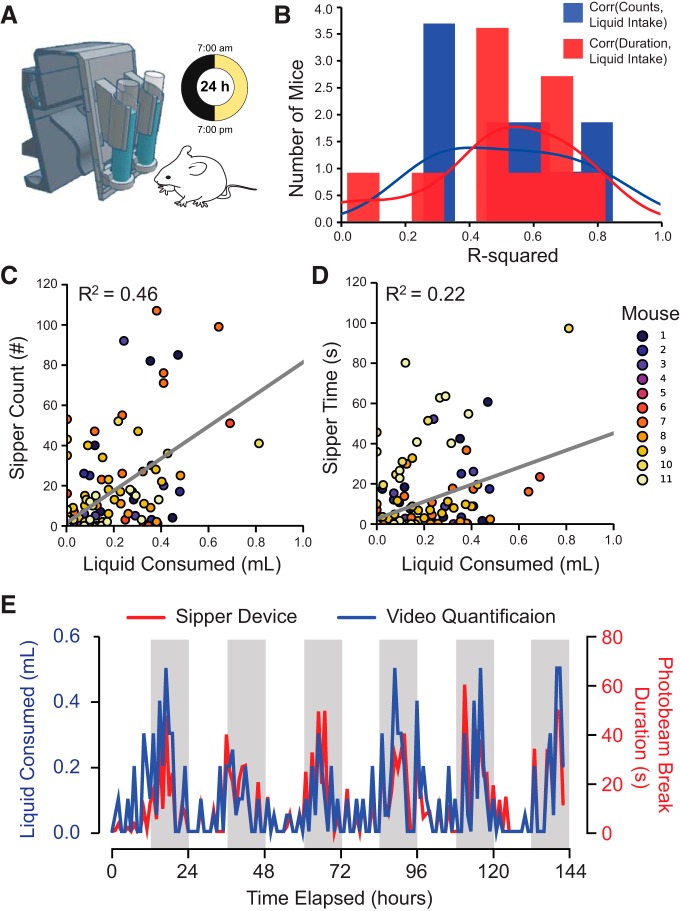
Functional validation of liquid consumption with the home-cage drinking monitor. ***A***, Schematic of the experimental setup. ***B***, Histogram depicting the distribution of *R* ^2^ values of the correlation between measurements (counts or duration) registered on the individual sipper devices with the volume of liquid consumed from the same device, as measured via time-lapse video. ***C***, ***D***, Across all 11 mice tested, sipper counts and durations of interactions with the sipper device weakly correlated with the amount of water consumed by visual quantification with time-lapse video. ***E***, Circadian rhythms in lick duration and volume of liquid consumed were evident over the 5 d of recording.

The sipper device provided the only source of drinking water, and the number and duration of photo-interrupter beam breaks on the sipper device and the volume of liquid consumed (detected via time-lapse video) were measured (Fig. 2*A*). The correlation between the volume ingested and sipper counts or duration varied across devices (sipper duration: *R* ^2^ = 0.02–0.89; sipper counts: *R* ^2^ = 0.26–0.85; Fig. 2*B*). Sipper counts were a better proxy for liquid intake with an average *R* ^2^ value of 0.46 (Fig. 2*C*) compared with the average *R* ^2^ value of 0.22 between sipper duration and liquid ingested (Fig. 2*D*).

The duration of sipper interaction registered on the sipper device showed the expected circadian rhythm, with drinking water consumption being greater during the dark cycle (78.3% of total sipper duration between 7:00 P.M. and 7:00 A.M.) relative to the light cycle (21.7% of total sipper duration between 7:00 A.M. and 7:00 P.M.). This pattern was confirmed with visual quantification of the liquid consumed via time-lapse photography (80.6% of liquid consumed in the dark cycle, *R* ^2^ between sipper duration and visual quantification = 0.63; Fig. 2*E*).

### Experiment 2

**Figure 3.**
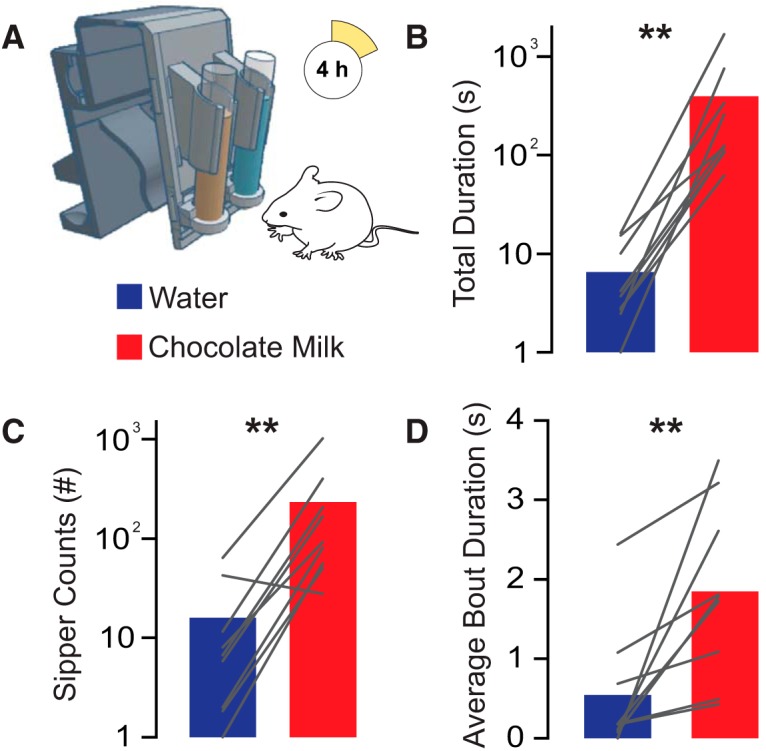
Two-bottle choice task with the home-cage drinking monitor. ***A***, Experimental schematic; mice had free access to two liquids in the drinking monitor device: water or chocolate milk. ***B–D***, All mice exhibited a clear preference for chocolate milk over water. Mice exhibited a longer total duration of sipper interactions (***B***), an increased duration of sipper interaction bouts (***C***), and an increased number of sipper approaches (***D***) for chocolate milk over water. **p < 0.01.

Preference for chocolate milk versus water was quantified using the sipper device as a two-bottle choice assay for a 4 h session (Fig. 3*A*). All mice exhibited a longer total duration of sipper interactions (reflecting total drinking time; mean water duration = 6.5 ± 2.0 s; mean chocolate duration = 396.5 ± 176.8 s; two-tailed Wilcoxon test, *p* = 0.0039; Fig. 3*B*), a greater number of sipper counts (reflecting the number of interactions with the sipper device; mean water counts = 15.9 ± 7.3; mean chocolate counts = 233.1 ± 105.1; two-tailed Wilcoxon test, *p* = 0.0078; Fig. 3*C*), and a longer average bout duration (total sipper duration/sipper count; mean water bout length = 0.55 ± 0.26 s; mean chocolate bout length = 1.85 ± 0.37 s; two-tailed Wilcoxon test, *p* = 0.0039; Fig. 3*D*) on the sipper containing chocolate milk relative to drinking water. Together, these results confirm the utility of the sipper device for studying liquid preference in a two-bottle choice assay.

### Experiment 3

**Figure 4.**
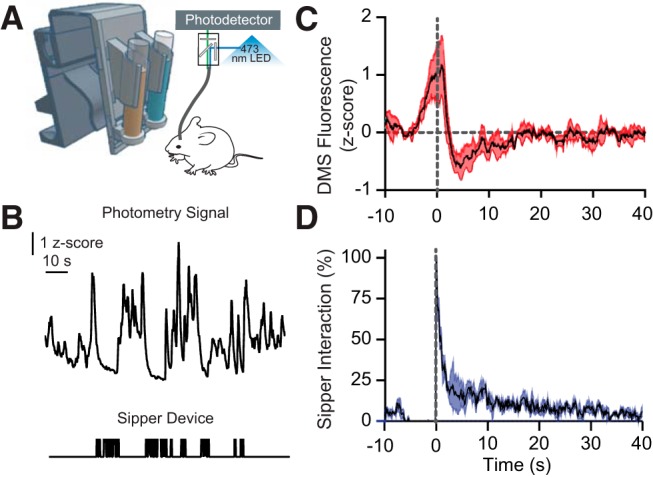
Integration of the drinking monitor with *in vivo* fiber photometry. ***A***, Schematic of the experiment; photo-interrupter beam breaks registered on digital Arduino pins triggered a TTL pulse to an *in vivo* fiber photometry system. ***B***, Representative raw trace of gCamp signal recorded in the DMS (top) and concomitant sipper interaction bouts (bottom). ***C***, Normalized gCamp traces were averaged across trials for all mice and were aligned to the onset of the lick bout. Black line, Mean; red, SEM. ***D***, The duration of sipper interactions is aligned to the onset of the lick bout; the mean probability of a sipper interaction is indicated in black, and SEM is depicted in blue. Both ***C*** and ***D*** are aligned to the onset of lick bout.

We confirmed that the sipper device can be successfully integrated with *in vivo* measurements of neural activity by allowing mice to consume chocolate milk from the sipper device while GCaMP fluorescence from the DMS was detected using a fiber photometry system (Fig. 4*A*,*B*). We chose theDorsomedial striatum (DMS) based on previous fiber photometry experiments that have shown that striatal neurons show a ramping activity before reward retrieval and are inhibited during reward consumption (London et al., 2018).

We confirmed that, relative to baseline, gCamp fluorescence in the DMS was significantly increased during approach to the sipper (*z* score = 0.89 SD above baseline), while fluorescence was significantly decreased during liquid ingestion from the sipper device (*z* score = −0.40 SD below baseline; Fig. 4*C*,*D*). This result demonstrates that the sipper device can be integrated with *in vivo* fiber photometry for measuring neural activation during liquid ingestion, and highlights the flexibility and application of open-source hardware for novel applications.

## Discussion

Here, we developed and validated a novel two-bottle choice sipper device that is useful for studying ingestive behavior in rodents. Our device has several advantages over traditional approaches to measuring liquid consumption. First, the device is compact, wireless, and battery powered, and has a battery life of >2 weeks, which enables high-throughput studies in vivarium caging. Second, our device provides data with a high temporal resolution, which allows for detailed analysis of circadian drinking patterns or analysis of drinking bout patterns and structure. Third, by dispensing with the need for capacitive sensors, our device is compatible with amplifiers or photodetectors used in studies to measure neuronal activity *in vivo*. Given that the device is based on photo-interrupters, animals are not required to “complete” an electric circuit for licks to be detected, which causes artifacts in electrophysiological recordings. Finally, our device is open source, and all design files and code are freely available. This allows for modifications to enable specific experiments, as well as easy integration with other recording systems, as we demonstrate with our fiber photometry recordings. Despite these strengths, there are some important limitations and areas of future development that must be kept in mind.

Our current design is a two-sipper configuration. However, there are several behavioral neuroscience paradigms in which it may be desirable to simultaneously monitor the intake of more than two liquids; for example, studies analyzing oral drug or ethanol discrimination. The microcontroller we use can monitor up to 13 sipper tubes with minimal modifications to the design and code, but these modifications would have to be performed by an end user. As a second limitation, our current design is built using 15 ml conical tubes as the liquid reservoir. While this supplied sufficient drinking water for at least 3 d, our experiments revealed that mice would frequently consume the entire volume of chocolate milk in the first 6 h of their dark cycle. Therefore, the rate of consumption is an important consideration for designing long-term experiments with the sipper device. Both of these issues can be overcome by modifying the construction of the device, which is readily achievable given the open-source design. In fact, the device has already been expanded by end users to include a three-sipper design, and a design with larger liquid reservoirs for use in rats (Frie and Khokhar, 2019).

The hardware used in the device also imposes some limitations that merit consideration. When an animal interacts with the sipper device, the photo-interrupters pick up interactions with millisecond resolution, but these interactions are not limited to licks. These interactions include the tongue of the animal, but can also include the snout or other body parts that break the infrared beam below the sipper. For this reason, we describe the device output as sipper counts or duration and not as “licks.” This limitation resulted from a design tradeoff that we made to build a wireless, home cage-compatible device versus a device that required specialized caging and tethered sensors, such as electrical lickometers. Both the sipper counts and duration were only moderately correlated with the volume of intake as measured by time-lapse photography (Fig. 2). This lack of tight correlation between lick behavior and liquid ingested has been well documented and is true of any traditional optical or capacitative lickometer (Schultz et al., 1989; Buxton and Allison, 1990; Ulman et al., 2008). However, there are two features of the sipper device that help to address this limitation. First, the reservoirs are constructed from 15 ml conical tubes with regular gradations. This makes visual inspection of liquid levels in the home cage straightforward, without the need to weigh bottles or otherwise remove traditional sipper tubes (which can be prone to spilling) from home cages. Second, the default code in the sipper device will write photobeam counts and duration to the microSD card every 10 s, unless there is an ongoing bout (defined as a broken photobeam) at the end of this 10 s interval. In this case, the duration and beam counts will be written at the end of the bout before proceeding to the next 10 s interval. This means that the time stamps can be inspected for outlying photobeam breaks, which could then be removed from the data as outliers. Therefore, while the device does not reliably detect individual licks or electronically measure liquid volumes, the device is still suitable for examining the duration, number, and pattern of consumption bouts (Figs. 2, 3).

A final feature is that the sipper devices are equipped with screens that display the duration and number of interactions on each sipper from outside of the home cage. Therefore, by inspecting the device screens, potential problems can be detected without interfering with the experiment. Finally, data retrieval requires the removal of SD cards and copying data to a computer, which can be time consuming if many devices are being used. In future versions, wireless communication chips could be added to the device to enable cloud-based data storage. Such a system would allow multiple users to access data with minimal interference, and would facilitate high-throughput and multisite experiments.

Beyond these specific concerns, the device is subject to limitations of all two-bottle choice assays. First, positional bias may affect drinking behavior (Gillespie and Lucas, 1957; Ackroff et al., 1993). Therefore, alternating the position of liquids in the two sipper tubes across time may be needed to control for this bias in preference assays. Second, the device currently requires animals to be singly housed, which may limit how many experiments can be run simultaneously due to space constraints and could introduce stress that may affect behavioral assays (Collins et al., 2012; Kamakura et al., 2016). However, as the device is open source, it could be modified to include additional sensors for discriminating multiple animals, such as RFID (radio-frequency identification) sensors that have been used in some commercial systems for this purpose (Galsworthy et al., 2005; Bains et al., 2016). While we have tried to optimize the device to prevent damage from mice and with long-term use as the goal, we found that mice sporadically chew on the sipper valves, which can interfere with the function of the device. These valves should be visually examined and replaced if mice chew on them.

In conclusion, we have developed a device for measuring liquid intake in home cages. The unique strengths of our system are that (1) it is wireless and compatible with vivarium home cages; (2) it is cost effective, costing <$100 to build each device; and (3) it is open source, allowing for modifications and flexibility for answering different experimental questions. The device logs interactions with two liquid sippers as detected by photo-interrupter beam breaks with high temporal resolution (Fig. 1). The device does not require the manual weighing of bottles and provides temporal resolution over timescales of several weeks without requiring a battery change, which makes the devices suitable for studying liquid preference and circadian biology or resolving long-term patterns of liquid intake (Figs. 2, 3). Our device was designed with scalability in mind: it requires no specialized equipment and is low cost and compact enough to allow for high-throughput experiments. We also provide a proof-of-concept validation that the device can be integrated with *in vivo* measurements of neuronal calcium activity (Fig. 4). These factors, combined with the open-source and off-the shelf nature of the device, ensure that it will be an accessible and useful tool for a variety of behavioral neuroscience experiments and studies of liquid ingestion.

## Synthesis

Reviewing Editor: Mark Laubach, American University Decisions are customarily a result of the Reviewing Editor and the peer reviewers coming together and discussing their recommendations until a consensus is reached. When revisions are invited, a fact-based synthesis statement explaining their decision and outlining what is needed to prepare a revision will be listed below. The following reviewer(s) agreed to reveal their identity: Zoe McElligott, Erin Calipari.

The following minor concerns were raised by the reviewers. These should be addressed before the manuscript can be considered for publication.

1 - Methods Experiment 1: is “falcon” tube a brand name?

Please clarify and revise accordingly.

2 - Results section “(Sipper Duration: R2= 0.02 to 0.83, mean = 0.51, Sipper Counts: R2= 0.26 - 0.85, Fig 2B)” is the dash supposed to be “to”?

Please revise to use either to or -.

3 - While not needed for the development of the method, I think it would be interesting to describe the DMS experiment a bit more, in particular in panel 4B there is both a red and black line and I do not know why (I assume that red is the water drinks and black is the chocolate milk? )

Please revise the text for panel 4B for clarity.

4 - The reviewers have also suggested that the authors should consider adding video analysis to confirm the sensitivity of their lickometer.

These additional data are not required for publication but would strengthen the manuscript.
